# The German 12‐Item Brief Form of the Cancer Behavior Inventory (CBI‐B‐D‐12): Factor Structure, Reliability, and Criterion Validity

**DOI:** 10.1002/pon.70313

**Published:** 2025-10-31

**Authors:** Juergen M. Giesler, Kathrin M. Gschwendtner, Christine Holmberg, Katrin Reuter, Joachim Weis

**Affiliations:** ^1^ Section of Health Care Research and Rehabilitation Research Medical Faculty and Medical Center Institute of Medical Biometry and Statistics University of Freiburg Freiburg Germany; ^2^ Department of General Internal Medicine and Psychosomatics Center for Psychosocial Medicine Heidelberg University Medicine Heidelberg Germany; ^3^ Institute of Social Medicine and Epidemiology Brandenburg Medical School Theodor Fontane Brandenburg an der Havel Germany; ^4^ Faculty of Health Sciences Joint Faculty of the Brandenburg University of Technology Cottbus‐Senftenberg the Brandenburg Medical School Theodor Fontane and the University of Potsdam Brandenburg Germany; ^5^ Joint Practice for Psychotherapy and Psycho‐Oncology (PPPO) Freiburg Germany; ^6^ Department of Self‐Help Research Comprehensive Cancer Center University of Freiburg Medical Center Freiburg Germany

**Keywords:** anxiety, coping, depression, fatigue, fear of progression, health‐related quality of life, self‐efficacy

## Abstract

**Background:**

The Cancer Behavior Inventory Brief Form (CBI‐B) allows assessing self‐efficacy for coping with cancer as a personal resource of patients facing a diagnosis of cancer and its treatment. While psychometric analyses of CBI‐B versions in other languages than English exist, the German version has not been analyzed more thoroughly in this respect yet.

**Aims:**

Against this background, we analyzed the factor structure, internal consistency, and criterion validity of the German 12‐item version of the Cancer Behavior Inventory Brief Form, the CBI‐B‐D‐12.

**Methods:**

Based on a pooled sample of *N* = 1034 cancer patients from various settings, we performed confirmative factor analyses, computed Cronbach's *α* and McDonald's *ω* for the 12‐item summary scale, and determined criterion correlations with measures of patients' health‐related quality of life, anxiety, depression, fear of progression, and fatigue.

**Results:**

With few adjustments, confirmative factor analysis revealed good fit of a 4‐factor model identifying the same dimensions of coping self‐efficacy as the original instrument (*Maintaining Independence and Positive Attitude*, *Participating in Medical Care*, *Coping and Stress Management*, and *Managing Affect*). With values of Cronbach's *α* and McDonald's *ω* being 0.89 and 0.88 respectively, estimates of the scale's internal consistency were good, and criterion correlations further supported its validity.

**Conclusions:**

The German 12‐item version of the CBI‐B represents a reliable measure of cancer patients' self‐efficacy for coping with cancer that is valid in terms of factorial structure and correlations with major distress and quality of life criteria. It may thus be used in clinical practice and psycho‐oncological research.

## 1 Background

Receiving a diagnosis of cancer confronts the individual with a series of challenging coping tasks. These may include experiencing varying levels of distress [1, 2], fears of recurrence [3] and of death [4], finding support from family and friends [5, 6, 7], acting upon medical information on cancer and its treatment [8], and participating in one's medical care and interacting with its providers [9, 10]. Furthermore, long‐term and late sequelae may need to be dealt with during survivorship [11, 12].

In conceptualizing the personal resources that may help cancer patients meet these challenges, the construct of self‐efficacy for coping with cancer (SECC) has received continuing interest in psycho‐oncological research, theory, and practice. Rooted in the more general theory of self‐efficacy [13], SECC is typically defined as a patient's confidence in his or her ability to perform specific coping behaviors in the context of cancer and its treatment [14]. Central to this conception is the by now well‐founded assumption that a patient's confidence in being able to perform a particular course of action helps initiate activities required to eventually achieve a goal desired in a specific context.

As an example, one may think of a cancer patient aspiring to retain a particular level of health‐related quality of life (HRQoL) while receiving chemotherapy. In this case, the patient might perhaps consider some recommended changes in dietary and activity behaviors in his or her daily routine. High confidence in being able to put these changes into effect then would, according to self‐efficacy theory, enable the patient to initiate these behaviors more easily and thus eventually succeed in retaining the desired level of HRQoL.

Given the pivotal role of SECC in respect to achieving personal goals, this example also illustrates the close connection between self‐efficacy theories and theories of self‐regulation and action [15]. At the same time, it stresses the importance of having psychometrically sound instruments available that allow one to assess cancer patients' SECC. Such instruments may help identify strengths and weaknesses in a patient's SECC that might subsequently be reinforced or improved through interventions aimed at reducing the patient's distress. In addition, such instruments may serve as outcome measures in interventions research and as measures of predictors of other outcomes like HRQoL or distress. Lastly, they would provide a basis for clarifying the possible role of SECC as mediators or moderators of relationships between treatment‐ and disease‐related variables on the one hand and psychosocial outcomes on the other.

The Cancer Behavior Inventory (CBI) represents one of the first measures of SECC [14]. Having undergone two revisions, it is currently available in version 3.0 [16], which encompasses 27 items covering coping behaviors in relation to cancer and its treatment. Since 2011, a psychometrically tested brief version, the CBI‐B, is also available [17]. Initially, this brief version consisted of 14 items taken from CBI version 2.0 [14], asking respondents to rate their confidence of being able to perform each of the presented coping behaviors on a 9‐point scale from “not at all” to “totally” confident. This version was by then reduced to 12 items, however, as exploratory and confirmatory factor analyses (EFAs and CFAs, resp.) suggested removing the original items no.’s 5 (“using denial”) and 14 (“remaining relaxed while waiting at least 1 h for my appointment”) [17]. Nevertheless, these analyses supported assuming the 12‐item version to cover four major domains of SECC: *Maintaining Independence and Positive Attitude*, *Participating in Medical Care*, *Coping and Stress Management*, and *Managing Affect*. Summing across a patient's item responses yields an overall score of his or her SECC. The internal consistency of this summary scale is good, with values of Cronbach's *α* ranging from 0.84 to 0.88 across three samples, and correlations with coping, distress and HRQoL characteristics further support its validity [17]. Although the factor analytic results might suggest computing domain‐specific subscale scores, this option has not been pursued in the questionnaire authors' research.

The CBI‐B has received wider international interest as reflected in the availability of translations in Arabic [18], Brazilian [19], Chinese [20], German [21, 22], Italian [23], Malay [24], Portuguese [25], and Turkish [26]. With values of Cronbach's *α* being above 0.80, the internal consistency of the overall scales of these versions is generally reported to be good, and correlations with distress criteria (anxiety, depression, etc.) or HRQoL characteristics support their validity [20, 23, 25]. Nevertheless, due to various methodological reasons (number of items included in factor analyses, type of factor analysis, criteria for retaining items of a hypothesized factor, or sample composition in regard to tumor site and stage, among others), the translated versions differ in their psychometric foundation to varying degrees limiting their comparability and that of findings of studies using them (see Supporting Information Table S1 for an overview of factor analytic results and selected sample characteristics as available from studies on translated CBI‐B versions [18, 19, 20, 23, 24, 25, 26]). While the CFA results for the Italian version come closest to the factor model for the original CBI‐B [17], the CFA of the Chinese version favors a single factor model, which is nevertheless in line with the recommended use of the CBI‐B as one‐dimensional measure [20]. In contrast, CFAs for the Portuguese version favored a 4‐factor model similar to the original CBI factor model, but along with considerations of possible cultural differences in item level responses led its authors to opt for an 11‐item summary score, as the fit of the respective 4‐factor model was only slightly, but significantly larger compared to a model including all 12 items [25]. Among the translated CBI‐B versions starting from the original 12‐item version, the Turkish CBI‐B differs most strongly from the original 4 factor structure in the loading pattern of its items, although its authors interpreted them as acceptable and also suggested using an overall summary score [26]. Starting from the earlier 14‐item CBI‐B, studies on the Arabic [18], Brazilian [19], and Malay [24] CBI‐B translations arrive at factor and scale structures that differ to an even greater extent from that of the original version. Whereas a 7‐item single scale score is suggested for the Arabic version [18], the Brazilian one was reduced to 10 items [19] consisting of two separate subscales with 6 and 4 items, resp. Providing only scarce information on its EFA, the Malay translation represents yet another variant with 12 items [24] built from four subscales differing in factor structure from that originally proposed [17]. Comparing these latter versions with the ones described before is even further complicated by the fact that they include those very two items that had been excluded from the original 12‐item CBI‐B in the course of its development [17]. Lastly, whereas the German CBI‐B had initially included all 14 items of the original American version, which were found to reflect the originally hypothesized 4‐factor structure [21], CFA of additional data later suggested the possibility of reducing it to 12 items [22] in line with the suggestions of Heitzmann et al. [17].

Considering the need to advance the comparability of international CBI‐B versions and the fact that the German version has only been evaluated psychometrically to a limited extent thus far, this study aims to analyze its validity and reliability on a broader basis asking:

- Will the original 4‐factor model proposed by Heitzmann et al. fit the German CBI‐B when restricted to only 12 of the original items,
- Will the overall 12 item scale be sufficiently reliable in terms of its internal consistency, and
- Will it correlate significantly and meaningfully with measures of distress like anxiety, depression and fear of recurrence, and with HRQoL characteristics?

For avoiding terminological ambiguity, the German 12‐item CBI version will hereafter be referred to as CBI‐B‐D‐12.

## 2 Methods

### 2.1 Design and Procedure

The present study uses data from five studies in which two of the authors (J.M.G., J.W.) were involved either as principle investigators [21, 27, 28] or as cooperating researchers [29, 30]. In each of these studies, the German 14‐Item CBI‐B served as a measure of SECC. With one exception, these studies included at least two, but no more than three measurement points for SECC and other patient reported outcomes. They addressed:

‐ changes of SECC either alone [21] (Study 1) or along with patient competencies during oncological inpatient rehabilitation [28] (Study 5),
‐ relationships between SECC, anxiety, depression, and fatigue in melanoma patients treated at a melanoma care center [29] (Study 2),
‐ effects of being given access to online illness narratives on patients with colorectal cancer in an RCT [30] (Study 3), or
‐ effects of a structured support group intervention on prostate and breast cancer patients, respectively [27] (Study 4).

In case a study repeatedly measured SECC, only SECC baseline data were included as predictors.

In each study, participants were treated in accordance with the declaration of Helsinki. They received information about the aims of the respective study, their right to decline participation or to withdraw from it without giving a reason or having to fear any disadvantages thereof, and data protection regulations being followed. Having provided consent, participants were included into a respective study and completed questionnaires as scheduled. For all studies except one, approval from the proper ethics committee at the respective institution was obtained. For Study 1, ethics approval had not been sought, as it was considered an exploratory study, which did not require ethics approval at that time.

### 2.2 Measures

#### 2.2.1 German CBI‐B Version

The original CBI‐B version [17] is based on the long version of the CBI, the CBI‐L 2.0 [14]. Following a forward‐backward procedure, two of the authors (J.M.G., K.R.) translated the 14 items of this CBI‐B version and its participant instructions into German in 2008 [21], which an independent native speaker then retranslated into English. Comparing the back‐translated and the original version by J.M.G., K.R. and J.W. suggested the translated version to be appropriate. Thereafter, a draft version of the questionnaire was produced and pre‐tested for comprehensibility in a small sample (*N* = 6). This resulted in few minor changes of item wordings, like choosing a more appropriate German term for “independence” (item 1, “Selbstständigkeit” instead of “Unabhängigkeit”, as the former expresses reliance on one's own abilities more directly). More importantly, the authors decided not to use the German equivalent of “negative feelings” (item 4), but to use the German term for “difficult” (enclosed in quotation marks) instead. This appeared to be more appropriate for addressing an individual patient's efforts to come to terms with conflicting and distressing emotions and also would avoid a possibly inappropriate negative evaluation of the feelings the patient experiences. Comments of patients of the pre‐test‐sample supported this decision. Another major change in wording related to “using denial” as in item 5. As experiences from the pre‐test suggested that a word‐for‐word translation (“Verleugnung einsetzen”) would create difficulty in understanding, the authors chose a wording that would read “Putting thoughts about cancer out of my mind at times” in English. As may be noted, this comes close to the rewording of that item, which is by now included in version 3.0 of the CBI and reads “putting things out of my mind at times” [16].

#### 2.2.2 Criterion Measures

Measures of cancer‐related distress and HRQoL that had been used in the studies forming the data base of the present analyses served as indicators of either concurrent or—as in Study 3—predictive validity of the CBI‐B‐D‐12. Being well established and available in psychometrically tested German versions, these measures will only be listed briefly here together with the construct they represent, that is, HRQoL: EORTC QLQ‐C30 [31]; fatigue: EORTC QLQ‐FA13 [32]; anxiety and depression: HADS [33], PHQ‐2 [34], and PHQ‐9 [35]; and fear of progression: FoP‐Q‐SF [36].

### 2.3 Statistical Methods

As indicated above, CBI‐B item responses were extracted from each of the five data sets along with information on participants' age and gender, which were collected in a comparable format across these data sets. Item responses as well as age and gender were then pooled into a single data file. Missing CBI item responses were imputed using the EM algorithm [37]. Subsequent data analyses included descriptive statistics for the CBI‐items, age, gender, and other major sample characteristics. In order to determine whether the original 4‐factor model [17] fit the CBI‐B‐D‐12, we ran CFAs [38] on its (12 items). As the traditional Maximum Likelihood (ML) approach to CFA has been shown to be problematic when analyzing ordinal data obtained from Likert‐type item response scales, as with the CBI‐B, we evaluated the hypothesized factor model using the by now recommended Weighted Least Square Mean Variance Adjusted (WLSMV) estimator [39]. Modification indices were used to improve model fit. The internal consistency of the CBI‐B‐D‐12 scale was estimated via Cronbach's *α* and also McDonald's ω, the latter being recommended when tau‐equivalence of items may not be given [40]. For determining criterion validity, we computed Pearson correlations between the CBI‐B‐D‐12 and the criterion measures within the five studies as available. Data analysis mainly used IBM SPSS, version 29. CFA was performed with the lavaan‐built JASP 0.95.2 software [41]. For CFA, CBI‐B item responses were treated as ordinal data. Therefore, item responses containing decimal places after having missing values imputed were rounded to the next integer.

## 3 Results

Table 1 presents major sociodemographic and medical characteristics of the combined sample, and the five subsamples included. Overall, patients were about 58 years old on average, 53% had received their cancer diagnosis within 12 months prior to study entry. Approximately 57% of the sample were female and 72% were either married or cohabitating. Of all participants, 37% had 12 or more years of formal education, 53% were employed. Tumor sites presented most often were breast and colorectal cancer, while approximately 16%–17% of patients had been diagnosed with prostate or skin cancer, respectively. Among those for whom tumor staging data were available, stages T2 (32%) and T3 (31%) were most frequent, 38% showed involvement of lymph nodes and about 23% had primary metastases.

**TABLE 1:** Sociodemographic and medical patient characteristics by study subsample and combined.^a^ ^,^ ^b^

Study subsamples	Study 1		Study 2		Study 3		Study 4		Study 5		Combined	
	(*N* = 135)		(*N* = 175)		(*N* = 212)		(*N* = 88)		(*N* = 424)		(*N* = 1034)	
Characteristic	*f*	%	*f*	%	*f*	%	*f*	%	*f*	%	*f*	%
Age (years: *M*, *SD*)	*n* = 133		*n* = 175		*n* = 212		*n* = 88		*n* = 420		*n* = 1028	
	54.6 (10.9)		58.5 (14.6)		54.1 (11.1)		60.4 (9.7)		61.5 (9.5)		58.5 (11.5)	
Diagnosed last 12 mths^c^	*n* = 134		*n* = 175		*n* = 212		*n* = 74		*n* = 397		*n* = 992	
	118	88.1	65	37.1	68	32.1	42	56.8	234	58.9	527	53.1
Gender^c^	*n* = 135		*n* = 175		*n* = 211		*n* = 88		*n* = 424		*n* = 1033	
Female	109	80.7	82	46.9	124	58.8	47	53.4	225	53.1	587	56.8
Male	26	19.3	93	53.1	87	41.2	41	46.6	199	46.9	446	43.2
Marital status	*n* = 135		*n* = 172		*n* = 167		*n* = 88		*n* = 421		*n* = 983	
Single	15	11.1	21	12.0			5	5.7	29	6.8	70	6.8
Married/cohabitating	93	68.9	124	70.9	167	78.8	75	85.2	290	68.4	749	72.4
Divorced/separated	17	12.6	12	6.9			5	5.7	59	13.9	93	9.0
Widowed	10	7.4	15	8.6			3	3.4	43	10.1	71	6.9
Education	*n* = 131		*n* = 168		*n* = 208		*n* = 86		*n* = 408		*n* = 1001	
9 years or less	37	27.4	81	46.3	20	9.4	23	26.1	124	29.2	285	27.6
10 years	45	33.3	44	25.1	78	36.8	33	37.5	132	31.1	332	32.1
12 years	11	8.1	18	10.3	17	8.0	8	9.1	51	12.0	105	10.2
13 years	38	28.1	25	14.3	93	43.9	22	25.0	101	23.8	279	27.0
Employment	*n* = 134		*n* = 170		*n* = 211		*n* = 88		*n* = 418		*n* = 1021	
Yes (full time or other)	68	50.4	71	40.6	117	55.2	59	67.0	237	55.9	552	53.4
No	66	48.9	99	56.6	94	44.3	29	33.0	181	42.7	469	45.4
Tumor site	*n* = 134		*n* = 175		*n* = 212		*n* = 88		*n* = 424		*n* = 1033	
Skin			175	100							175	16.9
Breast	81	60.0					47	53.4	145	34.2	273	26.4
Colon‐rectum	9	6.7			212	100			161	38.0	382	36.9
Prostate	5	3.7					41	46.6	118	27.8	164	15.9
Other	39	28.9									39	3.8
T							*n* = 85		*n* = 388		*n* = 473	*N* = 512^d^
T_1_							28	31.8	73	17.2	101	19.7
T_2_							37	42.0	126	29.7	163	31.8
T_3_							16	18.2	142	33.5	158	30.9
T_4_							3	3.4	33	7.8	36	7.0
X							1	1.1	14	3.3	15	2.9
N							*n* = 85		*n* = 398		*n* = 483	*N* = 512^d^
N_0_							52	59.1	200	47.2	252	49.2
N_1‐3_							27	30.7	168	39.6	195	38.1
X							6	6.8	30	7.1	36	7.0
Primary metastases					*n* = 212		*n* = 82		*n* = 387		*n* = 681	*N* = 724^d^
No					110	51.9	75	85.2	302	71.2	487	67.3
Yes					96	45.3	4	4.5	69	16.3	169	23.3
X					6	2.8	3	3.4	16	3.8	25	3.5
Secondary metastases							*n* = 74		*n* = 268		*n* = 342	*N* = 512^d^
No							72	81.8	193	45.5	265	51.8
Yes							2	2.3	75	17.7	77	15.0

^a^ Except otherwise noted, percentages for categorical variables represent *raw percent* with 100% equal to the total *N* given in the column main heading.

^b^ Empty cells: data not available; sub‐column *n*’s smaller than *N*’s given in column main headings due to missing values for sub‐column entries.

^c^ Percentages represent valid percent.

^d^ Here, raw overall percentages are based on the maximum possible *N* total as derived from the either two or three studies included (512 or 724, resp.).

**FIGURE 1:**
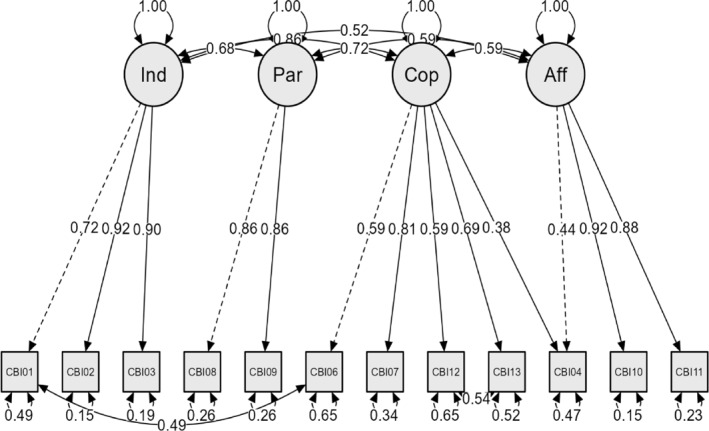
CFA 4‐factor model (χ^2^ (45) = 196.056, *p* < 0.001, CFI = 0.992, TLI = 0.988, RMSEA = 0.057 (90% CI: 0.049, 0.065), SRMR = 0.025). In order to enable comparison with previous research, item numbers match those of the original CBI‐B as published by Heitzmann et al. [17].

Figure 1 shows the CFA results for the hypothesized 4‐factor model of the CBI‐B‐D‐12 (loadings, factor intercorrelations, and error terms). The model identifies all 4 original SECC domains [17], its fit indices being χ^2^ (45) = 196.056 (*p* < 0.001), CFI = 0.992, TLI = 0.988, RMSEA = 0.057 (90% CI: 0.049, 0.065), and SRMR = 0.025. All correlations between factors are above 0.50, with the one between *Maintaining Independence and Positive Attitude* and *Coping and Stress Management* being highest (0.86). Subsequent item analyses of the CBI‐B‐D‐12 summary scale yielded comparable values of Cronbach's *α* and McDonald's ω, being 0.89 and 0.88, respectively.

Table 2 presents correlations between the CBI‐B‐D‐12 and criterion measures as available from the studies included. Where HRQoL was measured, mostly significant and medium (|r| ≥ 0.30) to high (|r| ≥ 0.50) correlations emerged between the CBI‐B‐D‐12 and the EORTC QLQ‐C30 functioning and symptom scales: higher SECC was associated with higher functioning and lower symptom scale scores. Within samples, the CBI‐B‐D‐12 correlated positively and most strongly with emotional functioning. In addition, it correlated significantly, highly, and negatively with depression in subsamples 2 to 5. Also, the CBI‐B‐D‐12 correlated significantly, moderately, and negatively with anxiety (study 2) as well as highly and negatively with fear of progression (studies 4 and 5). Measured solely in study 2, the three EORTC QLQ‐FA13 subscales correlated significantly and negatively with the CBI‐B‐D‐12 (−0.57 ≤ *r* ≤ −0.37; not in Table 2).

**TABLE 2:** Pearson correlations between baseline CBI‐B‐D‐12 and criterion measures of health‐related quality of life and distress in the 5 study subsamples of the present study.^a^

Study	Study 1	Study 2	Study 3^b^	Study 4	Study 5
Criterion variable	121 ≤ *n* ≤ 122	*N* = 175	105 ≤ *n* ≤ 107	*N* = 88	359 ≤ *n* ≤ 365
Health related quality of life					
Physical functioning	0.39^**^		0.39^**^	0.43^**^	0.25^**^
Role functioning	0.28^*^		0.26^*^	0.39^**^	0.19^**^
Emotional functioning	0.50^**^		0.47^**^	0.52^**^	0.44^**^
Cognitive functioning	0.28^*^		0.42^**^	0.52^**^	0.35^**^
Social functioning	0.43^**^		0.34^**^	0.39^**^	0.30^**^
Global health	0.41^**^		0.08	0.56^**^	0.33^**^
Fatigue	−0.36^**^		−0.31^**^	−0.47^**^	−0.31^**^
Nausea/Vomiting	−0.25^*^		−0.02	−0.01	−0.23^**^
Pain	−0.39^**^		−0.11	−0.35^*^	−0.24^**^
Anxiety		−0.47^**^			
Depression		−0.50^**^	−0.54^**^	−0.58^**^	−0.50^**^
Fear of progression				−0.62^**^	−0.49^**^

^a^ Empty cells: criterion variable not assessed.

^b^ As the design of study 3 had specified HRQoL (unlike depression) to be measured at a 2 weeks follow‐up for the first time, correlations involving HRQoL scales are truly predictive in this study. Also note that correlations in this column are based on control group subjects only in order to avoid otherwise possible confounding with potential treatment effects.

* *p* ≤ 0.01.

** *p* ≤ 0.001.

At the pooled sample level, correlations between the CBI‐B‐D‐12 and patient age (*r* = −0.07, *p* < 0.05) and gender (*r* = 0.11, *p* < 0.001, with male vs. female coded as 1 vs. 0) were significant, but small (not in Table 2). In subsample 4, however, men's CBI‐B‐D‐12 scores were notably higher than women's (*r* = 0.31, *p* < 0.005).

## 4 Discussion

The CFA results suggest the CBI‐B‐D‐12 represents the same four distinct domains of SECC as the original instrument, thus supporting its construct validity. With Cronbach's *α* and McDonald's *ω* being similarly high (0.89 and 0.88, respectively), the internal consistency of its 12‐item summary scale may be judged as good and comparable to estimates reported for the original CBI‐B and for the Chinese, Italian, and Turkish translations as based on the original 12‐item set [20, 23, 26]. Furthermore, criterion correlations with different distress measures and HRQoL were mostly substantial (|r| ≥ 0.30) and in the expected directions, thus supporting the criterion‐related validity of the instrument. This compares favorably to results of previous studies on the CBI‐B and its translations [17, 20, 23, 25, 26]. It also is in accordance with the role ascribed to SECC regarding the self‐regulation of action and emotion. Taken together, our findings thus suggest that the CBI‐B‐D‐12 is a reliable and valid measure of cancer patients' SECC.

In addition to this generally positive evaluation, a more detailed discussion of the presented factor model appears called for, however. As the χ^2^‐value of 4.358 is highly significant, it first should be noted that this indicator of model fit is highly sensitive to samples size, so that common additional indices need to be considered to evaluate model fit. Looking at these, one finds the values obtained for the CFI, the TLI, the RMSEA, and the SRMR to fall in regions defining either good (CFI > 0.97, SRMR < 0.05) or acceptable fit (RMSEA < 0.08) [38]. A second point for discussion concerns including correlated errors and one cross‐loading in the factor model. While this may raise criticism of being atheoretical [42], the results in the present case should be seen primarily as enabling a better understanding of the structure of the measure: Considering the content of the items involved it might be assumed that the error correlations of items 1 (maintaining independence) and 6 (maintaining work activity) and of items 12 (managing nausea) and 13 (coping with physical changes) each represent an albeit small, but semantically meaningful proportion of common variance that is unexplained by the other factors in the model. The same line of reasoning might be applied to the additional loading of item 4 (express negative feelings) on factor 3 (*Coping and Stress Management*). Given the brevity of the CBI‐B as a measure of SECC and its overall internal consistency, however, one would not assume these considerations to necessitate a revision of this instrument.

The foregoing discussion can be complemented by a brief look at the indicator reliabilities of the CBI‐B‐D‐12 items, which may be inferred by subtracting their respective error variances from 1. While most indicator reliabilities exceed the recommended minimum of 0.40 here, 2 items (no.'s 6 and 12) show error variances as high as 0.65 yielding indicator reliabilities of 0.35, which indicate they might be weaker indicators of the hypothesized underlying construct of *Coping and Stress Management*. Interestingly, these items in part performed similarly poorly in the studies analyzing the original, the Italian, and the Portuguese CBI‐B versions. Again, however, given the aforementioned reasons, one would not expect this to warrant revising the measure.

Comparing the factor structure established by the present analysis in its entirety to those reported by studies of other CBI‐B translations one finds it to be highly comparable to the American original. In contrast, some discrepancies emerge when comparing this model to those of other translated versions. In the cases of the Italian and the Portuguese CBI‐B single items loading on a factor different from the analysis of the original version might be considered less crucial, whereas the factorial structures of the Chinese and the Turkish version differ to a larger extent from those others just referred to. However, insofar as all these versions except for the Portuguese one followed the established practice of computing a 12‐item SECC summary score and—including the Portuguese one—reported substantial and meaningful criterion correlations, there still appears to be an acceptable degree of comparability at this level. In contrast, the summary score of the Arab, Brazil, and Malay CBI‐B versions only allows measuring SECC across a restricted range of domains. An important task for future research may thus lie in clarifying whether and how measuring SECC at specific domain levels would offer additional benefit.

A last issue to be discussed in this context refers to possible explanations of differences in factor structures reported for the CBI‐B and its translations. Obviously, there are several determinants to be considered here, with sample composition in regard to tumor site, disease stage or treatment modality coming to mind first, as they may present quite different coping tasks and experiences to patients, which might then affect parameters like item difficulty, standard deviation, or item‐intercorrelations. Beyond condition and treatment related characteristics, socio‐cultural factors or the social organization of cancer care will also shape patients' beliefs in their SECC or, at an even more fundamental level, how the “task” of “coping with cancer” is being constructed. For illustrating possible effects of sample composition it might be noted that for example the Portuguese CBI‐B study included only breast cancer patients receiving chemotherapy, whereas the Turkish CBI‐B study recruited solely cancer patients undergoing radiotherapy, and the Chinese CBI‐B study included predominantly patients with more advanced disease (stages III or IV). Not surprisingly then, comparing the item means of “coping with physical changes” across these studies (6.71 (SD = 1.43), 5.12 (2.58), and 6.28 (2.08), resp.) reveals differences of the size of approximately half a SD. In contrast, an example of cultural effects on a coping behavior like “expressing negative feelings about cancer” is also presented in the study on the Portuguese CBI‐B as well, whereas a discussion of cultural traditions that focus less on concepts central to theories of SECC like agency and personal control [43] provides an example of cultural effects operating at a more fundamental level. As a consequence of these considerations, future research on translated versions of the CBI‐B as a measure of SECC should pay even closer attention to these context factors as they affect conceptualization and measurement at different stages and levels. This may greatly help advance cross‐cultural research on SECC. Regarding the CBI‐B‐D‐12, its structural invariance, convergent validity, and sensitivity to change would also represent important topics for future research, although initial studies on the latter two are already available [28, 44].

### 4.1 Implications

Turning to the practical implications of its findings, this study points to the potential usefulness of the CBI‐B‐D‐12 in clinical and research contexts as already referred to in the introduction and similar to conclusions drawn in other CBI‐B studies. Based on further supplementary psychometric research, the CBI‐B‐D‐12 may successfully be used for screening cancer patients' coping resources in clinical settings and help plan and evaluate interventions aimed at advancing their SECC and alleviating distress. Interventions research using the instrument as an outcome will certainly also help achieve this goal.

### 4.2 Strength and Limitations

Strength of the present analyses include the large sample size, the multi‐center basis, and the coverage of a wider range of tumor sites. Major limitations result from not including patients from acute care settings, patients suffering from other cancers (e.g., lung or gynecological cancer, and lymphoma), and long term survivors. Also, the secondary character of the present analyses implies that relevant medical characteristics were not available for portions of the sample, thus limiting the possibility of determining potential restrictions for generalizability.

## 5 Conclusion

The CBI‐B‐D‐12 proved to be a reliable and valid instrument covering the same domains as the original measure and, with only minor discrepancies as long as the summary 12‐item scale is concerned, the translations in Chinese, Italian, Portuguese and Turkish. For the sake of comparability, it should thus be preferred over the earlier 14‐item version. It may be used in clinical settings for screening and intervention purposes, in interventions research, and in research addressing the role of SECC in self‐regulation of emotion and action.

## Author Contributions

J.M.G. and J.W. study conceptualization and data analysis, drafting of manuscript, conduct and data analysis of studies 1, 3, 4, and 5, K.G. primary data analysis of study 4, C.H. conceptualization and conduct of study 3, K.R. conceptualization and conduct of Study 2. All authors participated in finishing the manuscript and approved the final version.

## Ethics Statement

Available ethics approval had been provided by the Ethics Committee University of Freiburg (Study 2 see NCT00963261; Studies 4 and 5: numbers 151/13 and 359/12, resp.) and the Ethics Committee Charité Universitätsmedizin Berlin (Study 3: EA4/053/12).

## Consent

Informed consent was obtained from all participants included in the study.

## Conflicts of Interest

The authors declare no conflicts of interest.

## Supporting information


**Table S1:** Major sample characteristics and results of previously published studies analyzing the factor structure of CBI‐B translated versions.

## Data Availability

The data on which the present analyses are based will be available from the corresponding author upon reasonable request.
