# Supplementary material for: The German 12‐Item Brief Form of the Cancer Behavior Inventory (CBI‐B‐D‐12): Factor Structure, Reliability, and Criterion Validity
Source: Psychooncology. 2025 Oct 31;34(11):e70313. doi: 10.1002/pon.70313 (PMC12577206; doi:10.1002/pon.70313)
Supplement: Supplementary file 1 — Table S1: Major sample characteristics and results of previously published studies analyzing the factor structure of CBI‐B translated versions. [file PON-34-e70313-s001.pdf]

Supplementary Table S1: Major sample characteristics and results of previously published studies analyzing the factor structure of CBI-B translated versions.

**CBI-B-Version**	**Original**	**Chinese**	**Italian**	**Portuguese**	**Arab: All**	**Arab: Breast**	**Brazilian**	**Malay**	**Turkish**
Year of publication	**2011**	**2021**	**2019**	**2021**	**2018**		**2024**	**2020**	**2017**
Analysis N	370	386 (CFA)	216	115	438	168	140	168	143
% Breast Cancer	46	10	44	100	38	100	36	100	20
% Male	36	54	34	0	24	0	33	0	66
Age (Yrs, Md, Range or M±SD)	73, n.a.	54.8±12.7	62, 35 - 86	52.7±10.2	48.2±12.8	48.2±8.8	60.1±15.5	51.4±10.8	57.2±17.2
Number of items analyzed	12	12	12	12	12	14	14	14	12
Number of Items retained	12	12	12	11	7	7	10	12	12
Type of Analysis	CFA	CFA (after EFA)	CFA	CFA	EFA	EFA	EFA	EFA	EFA
**Factors and Items**									
**Independence and Positive Attitude (I)**									
CBI_01. Maintaining independence	I	I	I	I	F1	F1	F1	F3	F1
CBI_02. Maintaining positive attitude	I	I	I	I	F1	F1	F1	F1	F1
CBI_03. Maintaining sense of humor	I	I	I	I	F1	F1		F1	F1
**Participating in Medical Care (P)**									
CBI_08. Participating in Tx decision	P	I	P	P	F1	[F3]	F2	F2	F3
CBI_09. Asking physicians questions	P	I	P	P	[F1]	[F3]		F3	F3
**Coping and Stress Management (S)**									
CBI_06. Maintaining work activity	S	I	I	S	F1	F1	F1	F3	F1
CBI_07. Relaxed receiving treatments	S	I	S	S	F1	F1		F1	F3
CBI_12. Managing nausea and vomiting	S	I	S	S	[F1]	[F1]	F1		F2
CBI_13. Coping with physical changes	S	I	S	S	F1	F1		F2	F1
**Affect Management (A)**									
CBI_04. Expressing negative feelings	A	I	A	<A>	[F2]	[F2]	F2	F4	F2
CBI_10. Seeking consolation/support	A	I	A	A	[F2]	[F4]	F2	F4	F4
CBI_11. Sharing feelings of concern	A	I	A	A	[F2]	[F4]	F2		F2
**Items excluded by Heitzmann et. al. 2011**									
CBI_05. Denial/Put things out of mind	-	-	-	-	-	[F2]	F1	F2	-
CBI_14. Remain relaxed when waiting	-	-	-	-	-	F1	F1	F2	-

Note: n.a.: not available. Capital letters I, P, S, and A indicate to which CBI-B factor an item was assigned based on its loadings, with angle brackets indicating removal of a substantially loading item to improve model fit. Labels F1 to F4 indicate on which of up to 4 extracted factors an item loaded in a study, with square brackets indicating items not retained as their loadings did not meet the study authors' criteria for including them in defining a factor (i.e. loading ≥ .30, no cross-loading, or minimum number of substantially loading items per factor). Completely empty cells indicate items having been excluded for the same reasons, but without giving exact information on the size of their loadings.
